# Quantum Phase Transitions in Graphene Coupled to a Twisted WSe_2_ Moiré Ferroelectricity

**DOI:** 10.1002/adma.202514744

**Published:** 2025-10-22

**Authors:** Budhi Singh, Yasir Hassan, Nasir Ali, Santhosh Durairaj, Jimin Jang, Tien Dat Ngo, Jyoti Saini, Muhammad Sabbtain Abbas, Kenji Watanabe, Takashi Taniguchi, Min Sup Choi, Subhasis Ghosh, Taesung Kim, Hyung Mo Jeong, Sungjoo Lee, Won Jong Yoo, Pawan Kumar Srivastava, Changgu Lee

**Affiliations:** ^1^ SKKU Advanced Institute of Nanotechnology (SAINT) Sungkyunkwan University Suwon 16419 South Korea; ^2^ Department of Materials Science and Engineering Chungnam National University Daejeon 99 South Korea; ^3^ School of Mechanical Engineering Sungkyunkwan University Suwon 16419 South Korea; ^4^ School of Physical Sciences Jawaharlal Nehru University New Delhi 110067 India; ^5^ Insitute of Applied Physics Seoul National University Seoul 08826 South Korea; ^6^ Centre for Advanced Studies in Physics GC University Lahore 54000 Pakistan; ^7^ National Institute for Materials Science Namiki 1‐1 Tsukuba Ibaraki 305‐0044 Japan; ^8^ Center for 2D Quantum Heterostructures Institute for Basic Science (IBS) Sungkyunkwan University (SKKU) Suwon 16419 Republic of Korea; ^9^ Department of Physics National University of Singapore Science Drive 3 Singapore 117542 Singapore

**Keywords:** Dirac point, metal‐insulator transition, moiré ferroelectricity, quantum phase transitions, sublattice symmetry breaking, twisted WSe_2_, t‐WSe_2_/graphene field‐effect transistor

## Abstract

Sublattice symmetry in graphene governs its Dirac semimetal behavior, where electrons exhibit linear dispersion, limiting its potential for technological applications. Here, moiré ferroelectricity in twisted WSe_2_ (*t*‐WSe_2_) is exploited to break graphene's sublattice symmetry, inducing a metal‐to‐insulator transition (MIT) near room temperature. The periodic polarization domains in *t*‐WSe_2_ imprint an electrostatic potential onto graphene, breaking its sublattice symmetry and leading to the emergence of a local Dirac point, as observed in the transfer characteristics of a *t*‐WSe_2_/graphene field‐effect transistor. Temperature‐dependent transport measurements reveal multiple MIT points at relatively high temperatures, attributed to the room‐temperature ferroelectric polarization in *t*‐WSe_2_. Furthermore, A distinct metallic phases is identified exhibiting *T* ^2^ and linear‐*T* dependent longitudinal resistance under electrostatic doping, indicative of Fermi‐liquid and non‐Fermi‐liquid metallic behavior, respectively. Finally, finite‐size scaling analysis of *R*_xx_ near the MIT points indicates continuous quantum phase transitions near room temperature, establishing moiré ferroelectricity as a pathway for engineering quantum electronic phases of monolayer graphene at ambient conditions.

## Graphical abstract

Moiré ferroelectricity in twisted WSe_2_ (*t*‐WSe_2_) breaks graphene's sublattice symmetry, inducing a room‐temperature metal‐insulator transition. Coupling graphene with ferroelectric domains of *t*‐WSe_2_ creates local Dirac points and metallic phases with Fermi‐liquid and non‐Fermi‐liquid behavior. Scaling analysis confirms continuous quantum phase transitions, establishing a route totunable quantum phases in graphene at ambient conditions.

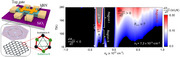

## 1 Introduction

Phase transitions represent fundamental reorganizations of a system's ground state, driven by symmetry breaking and collective excitations, where microscopic interactions lead to the emergence of macroscopic order and distinct physical properties.^[^ ^1^, ^2^, ^3^ ^]^ In quantum systems, phase transitions often arise from competition between electronic correlations, topology, and quantum fluctuations, leading to exotic states such as superconductivity, spin liquids, and topological phases, often observed at temperatures near that of liquid helium.^[^ ^4^, ^5^ ^]^ The ability to tune such phase transitions through external fields, strain, or moiré engineering not only deepens our understanding of many‐body physics but also drives the development of quantum technologies. Moiré engineering has emerged as a powerful approach to realizing correlation‐driven phenomena such as superconductivity, quantum magnetism, and metal‐insulator transitions (MITs).^[^ ^6^, ^7^, ^8^, ^9^, ^10^, ^11^, ^12^, ^13^, ^14^ ^]^ However, despite its potential, the development of quantum technologies based on twistronics remains limited by the requirement of liquid helium temperatures. Among various advancements in twistronics, ferroelectricity driven by broken inversion symmetry in certain van der Waals (vdW) materials stands out as a promising platform, as their ordered polarization domains can persist even at room temperature.^[^ ^14^, ^15^, ^16^, ^17^, ^18^, ^19^ ^]^ This robustness opens the door to electronic functionalities without the requirement for extreme cooling, paving the way for more practical device applications.

Graphene, a widely studied vdW material, presents an interesting challenge in this context. Its intrinsic sublattice symmetry results in a Dirac semi metallic behavior with relativistic electrons, making it difficult to induce an insulating state, a key limitation for its technological deployment.^[^ ^20^ ^]^ Moreover, the investigation of MIT in 2D materials is particularly relevant because earlier theoretical models, such as the Mermin‐Wagner theorem and Anderson localization, suggest that metallic behavior should not be achievable in such systems.^[^ ^21^, ^22^, ^23^ ^]^ In this context, we carefully designed twisted WSe_2_ (*t*‐WSe_2_)/graphene heterostructures encapsulated between *h*‐BN layers, where the effect of a moiré periodic potential induced by *t*‐WSe_2_ enables more effective tuning of graphene's electronic properties. The moiré pattern arises from a small twist angle (*θ*) between the WSe_2_ monolayers, and the associated moiré potential originates from the electrostatic potential generated by ferroelectric domains in *t*‐WSe_2_.^[^ ^24^, ^25^ ^]^ Consequently, the spatially varying electric field from ferroelectric domains can break graphene's sublattice symmetry, leading to enhanced tunability of its electronic transport characteristics. Since ferroelectric domains remain stable at room temperature, their influence on graphene's sublattice symmetry is also expected to persist under ambient conditions.

Furthermore, the *θ* in *t*‐WSe_2_ provides a systematic means of controlling ferroelectric domains and thus the moiré superlattice potential.^[^ ^15^ ^]^ This tunability makes *t*‐WSe_2_ a particularly versatile and highly controllable platform for probing how ferroelectric domains perturb the Dirac quasiparticles in graphene, an advantage not readily achievable with conventional 2D ferroelectrics such as *α*‐In_2_Se_3_, SnS, and CuInP_2_S_6_.^[^ ^26^ ^]^ For comparison, in *h*‐BN/graphene vdW heterostructures, local Dirac points arise from sublattice symmetry breaking caused by precise alignment between graphene and *h*‐BN. This alignment produces a periodic moiré potential from lattice mismatch, which in turn generates minibands and secondary Dirac points near the moiré Brillouin zone edges.^[^ ^27^ ^]^

In this article, we first provide direct experimental evidence of sublattice symmetry breaking in graphene proximitized by a moiré ferroelectric material, *t*‐WSe_2_. We then demonstrate its significant impact on MITs behavior at ambient conditions. Furthermore, we observe different metallic phases characterized by *T^2^* and linear‐*T* dependence of the longitudinal resistance (*R*_xx_), which we interpret through experimentally verified scattering mechanisms. Finally, we reveal the quantum nature of this phase transition via finite‐size scaling analysis.

## 2 Results and Discussion

**Figure 1:**
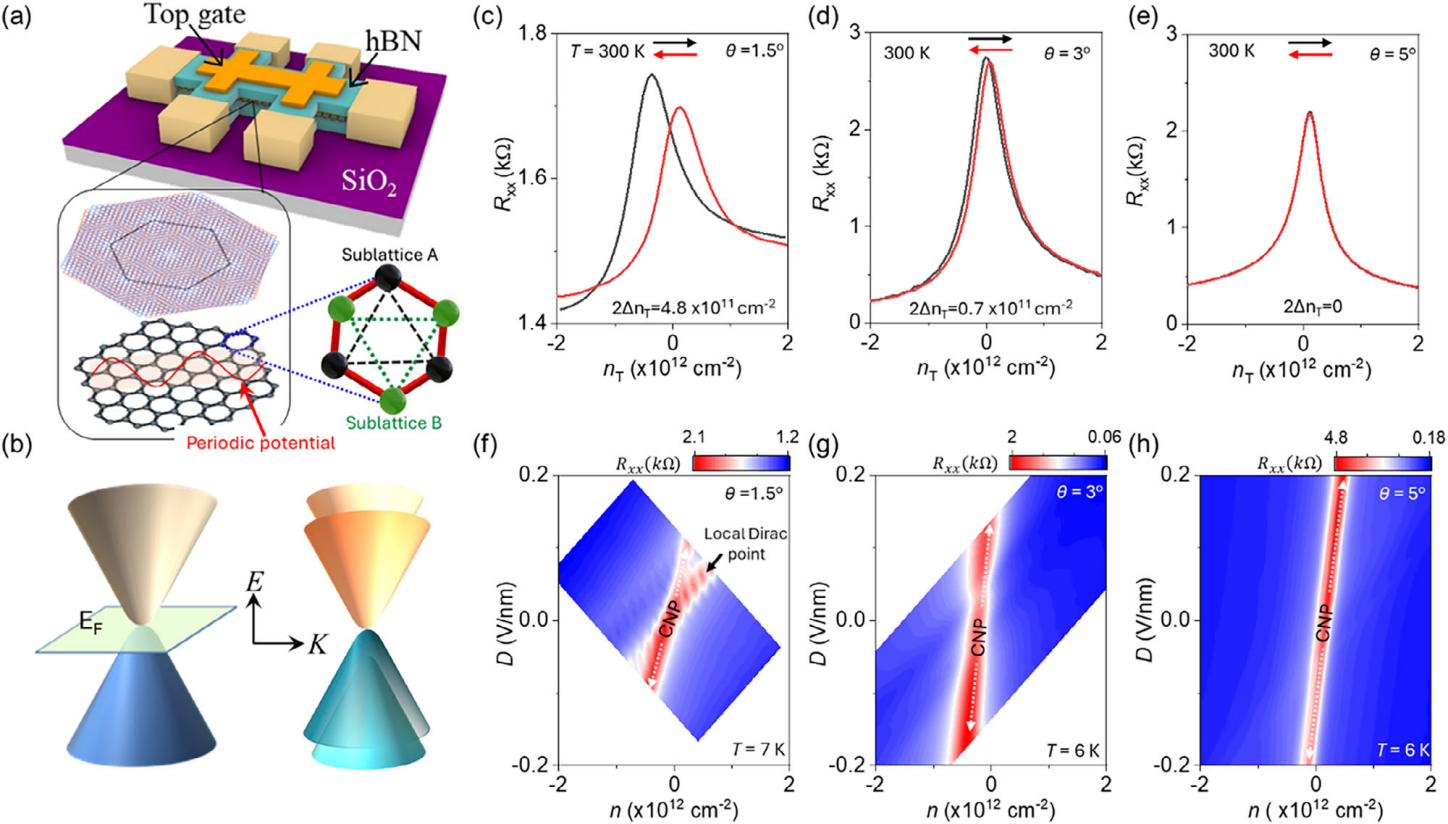
Sublattice symmetry breaking of graphene: a) Schematic of the dual‐gated *h*‐BN encapsulated *t*‐WSe_2_/graphene vdW moiré heterostructures. The graphene layer is positioned beneath the *t*‐WSe_2_, experiencing its periodic moiré potential, as illustrated by the red curve. Here, 290 nm SiO_2_ serves as the dielectric for the *V*_BG_, while 30 nm *h*‐BN serves as the dielectric for the *V*_TG_. b) Schematic for the sublattice symmetry breaking in the monolayer graphene. c–e) Twist controlled ferroelectricity in *t*‐WSe_2_/graphene vdW heterostructures for *θ* = 0°, 3° and, 5° at room temperature. Here, *θ* signifies twist angle between bilayer WSe_2_. The hysteresis in the forward (black) and backward (red) scans of electrostatic doping through *V*_TG_ could be attributed to ferroelectric switching in twisted WSe_2_. Inset shows the ferroelectricity‐induced net carrier density, 2*Δn*_T_ in *t*‐WSe_2_/graphene vdW heterostructures. f–h) The 2D color plot of *R*_xx_ as a function of electrostatic doping, *n*, and displacement field, *D* for *θ* = 0°, 3° and 5° *t*‐WSe_2_/graphene vdW heterostructures at low temperature. The emergence of two peaks in transfer characteristics of graphene FET is a signature of sublattice symmetry breaking in graphene. These peaks are labeled as local Dirac point and the charge neutrality point (CNP) in the 2D color plots.

Sublattice symmetry breaking of graphene: **Figure** **1**a shows the schematic of the dual‐gated *h*‐BN encapsulated *t*‐WSe_2_/graphene vdW heterostructures used to demonstrate sublattice symmetry breaking in graphene through electrical transport measurements (*see* methods).^[^ ^15^ ^]^ This vdW heterostructure was intentionally designed to induce a periodic potential in monolayer graphene, coupled with the moiré superlattice of *t*‐WSe_2_. In this system, moiré ferroelectricity emerges due to the periodically arranged up and down ferroelectric domains in *t*‐WSe_2_, which imprints an electrostatic potential onto the adjacent monolayer graphene, as illustrated in Figure 1a.^[^ ^25^ ^]^ The spatially varying electric field at the *t*‐WSe_2_/graphene interface break sublattice symmetry by introducing an energy difference between the two inequivalent sublattices. As a consequence, the initially degenerate Dirac cones at the *K* and *K'* points of graphene's Brillouin zone become non‐degenerate, lifting valley degeneracy. This suppression of degeneracy disrupts the relativistic electronic behavior of graphene, significantly modifying its band structure and quenching many of its characteristic unusual electronic properties (Figure 1b). In order to test this conjecture, we monitored the dual gated electrical transport characteristics of our devices under various twist angles between WSe_2_ bilayers, which ultimately dictates the ferroelectric regimes. Figure 1c–e shows the twist‐angle‐controlled ferroelectricity in WSe_2_ bilayers, revealed by the hysteresis observed in the forward and reverse top‐gate voltage (*V*_TG_) scans of the *t*‐WSe_2_ coupled graphene field‐effect transistors (FETs). The ferroelectricity in *t*‐WSe_2_ is maximum when *θ* = 1.5°, and vanishes completely for *θ* > 3°.^[^ ^15^ ^]^ This variation corresponds to moiré length scale‐controlled ferroelectric dynamics (0° < *θ* < 3°), while the loss of ferroelectricity beyond 4° is associated with the transition from commensurate to non‐commensurate moiré patterns.^[^ ^15^, ^17^, ^19^, ^28^, ^29^ ^]^

2D color plots of *R_xx_* of graphene FETs of same devices as a function of electrostatic doping, *n*, and vertical average displacement field, *D* at low temperature of ≈6 K are summarized in Figure 1f–h. Here, *n* and *D*, are expressed as: $n=\frac{1}{e}\;\left(D_{bg}+D_{tg}\right)$ and $D=\frac{1}{2}\;\left(D_{bg}-D_{tg}\right)$, where $D_{bg}=\frac{\varepsilon_{SiO_{2}}V_{BG}}{d_{SiO_{2}}}\;$ and $D_{tg}=\frac{\varepsilon_{hBN}V_{TG}}{d_{hBN}}\;$ represent the displacement fields due to back gate voltage (*V*_BG_) and *V*_TG_, respectively. ε_SiO2(_ *_h_*_‐BN)_ = 3 and *d*_SiO2(_ *_h_*_‐BN)_ represents dielectric constant and thickness of SiO_2_ and *h*‐BN, respectively. The *R*_xx_ peak corresponding to CNP of graphene is observed for all the devices. In addition to CNP, other prominent peak in the *R*_xx_ arises from broken sublattice symmetry that lifts the degeneracy of the Dirac points. We refer to this additional peak in the transfer characteristics of the graphene FET as the local Dirac point. The local Dirac point peak is the most prominent for *θ* = 1.5° (Figure 1f) compared with *θ* = 3.0° (Figure 1g; Figure S1, Supporting Information). At higher twist angle, *θ* = 5.0°, no such peak is observed because there are no commensurate ferroelectric domains (Figure 1h).^[^ ^15^ ^]^

Conceptually, these devices also anticipate additional peaks at *n* = 7.3 × 10^11^ cm^−2^, 3.0 × 10^12^ cm^−2^ and 8.0, × 10^12^ cm^−2^ for *θ* = 1.5°, 3° and 5° corresponding to the half‐filling of moiré subband in *t*‐WSe_2_. This is because the carrier density for the full‐filling *t*‐WSe_2_ moiré subband, assuming two holes per unit cell, is given by $n_{s}=\frac{2}{A}$, where $A=\frac{a^{2}\sqrt{3}}{4(1-\cos(\theta ))}$, is the area of *θ*‐dependent moiré unit cell. Here, *a* = 0.328 nm is the lattice constant of WSe_2_.^[^ ^30^ ^]^ However, no peaks corresponding to half‐filling of moiré superlattice are observed in the transfer characteristics of graphene FETs coupled to *t*‐WSe_2_. This is due to the large energy difference (>0.5 eV) between the graphene Dirac point (≈4.6 eV) and the WSe_2_ band edges (≈5.31 eV) (Figure S2, Supporting Information). The *V*_TG_ can only shift the Dirac point maximum up to 0.1–0.3 eV as per our device capacity before the breakdown of the *h*‐BN dielectric. Consequently, *t*‐WSe_2_ remains undoped by *V*_BG_, and carriers from *V*_BG_ only enter the monolayer graphene.

**Figure 2:**
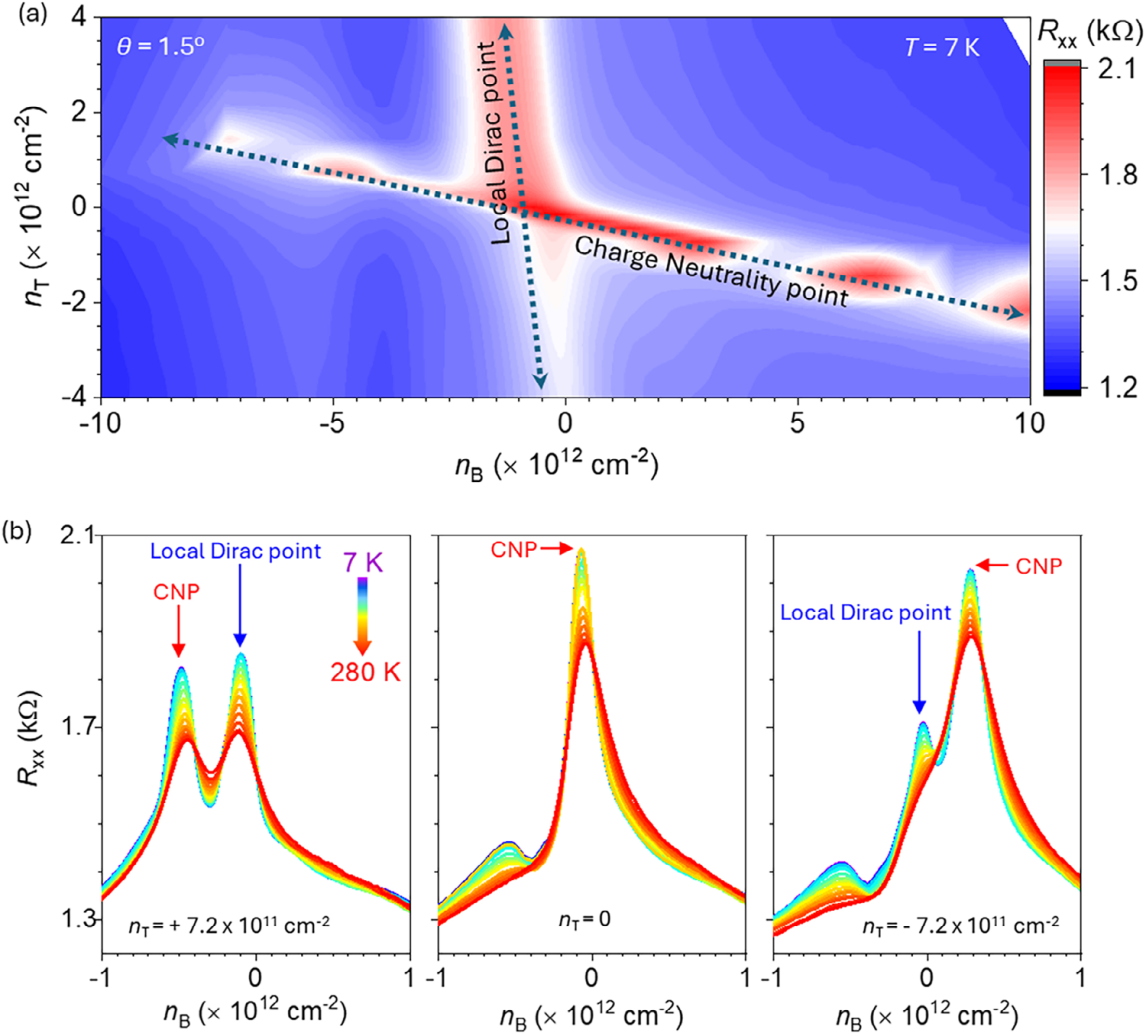
Metal‐to‐insulator transition in graphene: a) 2D color map of the dual‐gated electronic transport measurements, i.e.*, R_xx_* vs *n_B_* vs *n_T_* of the graphene FET coupled with ferroelectric *t*‐WSe_2_ for twist angle, *θ* = 1.5° at 7 K. The colors in the 2D map indicate the presence of two peaks in the *R_xx_* vs *n_B_* curve when measured at constant *n_T_*. These peaks are due to the charge neutrality point of monolayer graphene and the local Dirac point due to sublattice symmetry breaking in graphene. b) Temperature‐dependent *R_xx_* vs *n_B_* curves over the temperature range from 7 to 280 K corresponding to electrostatic doping *n_T_* of +7.2 × 10^11^ cm^−2^, 0, and ^−^7.2 × 10^11^ cm^−2^, respectively.

Metal‐insulator transitions: We deliberately choose the device with *θ* = 1.5° where the signature of broken sublattice symmetry of graphene is prominent. **Figure** **2**a shows the dynamical shift of the *R*_xx_ peaks with respect to *V*_BG_ and *V*_TG_ at 7 K. The 2D color map of the dual‐gated electronic transport measurements at 7 ‐K indicates the presence of two peaks in the *R*_xx_ vs *n*_B_ ($=\frac{\varepsilon_{SiO2}V_{TG}}{e\;d_{SiO2}}\;$) curve when measured at constant, *n_T_* (= $\frac{\varepsilon_{hBN}V_{BG}}{e\;d_{hBN}}$). The *R*_xx_ peak corresponding to the CNP of graphene shifts from the electron regime (negative *n_T_*) to the hole regime (positive *n_T_*). This shift occurs because the *t*‐WSe_2_ bilayer together with top *h*‐BN behaves like a gate dielectric for the monolayer graphene. Consequently, the shift in the Fermi energy of monolayer graphene with *n_T_* causes corresponding shifts in the CNP peak of *R*_xx_ with *n_B_* at various *n_T_*. It has also been observed that *n*_T_ modulates the commensurate ferroelectric domains, as observed through the slight shift of the local Dirac point peak with *n*_T_. Therefore, the dynamical interplay of ferroelectric commensurate domains in *t*‐WSe_2_ and delocalized electrons in the dispersive band of monolayer graphene with *n*_T_ reveals strong coupling between graphene's electronic properties and the ferroelectric order in *t*‐WSe_2_. This coupling enables electric‐field control over the electronic structure at the *t*‐WSe_2_/graphene interface, consequently breaking the sublattice symmetry of graphene and leading to anomalous temperature‐dependent transport properties. In this context, we investigated the electrical transport characteristics of the *t*‐WSe_2_/graphene vdW heterostructure for *θ* = 1.5° at various temperatures.

Figure 2b shows the *R*_xx_ vs *n_B_* curves over the temperature range from 7 to 280 K at fixed *V*_TG_ corresponding to electrostatic doping *n_T_* of +7.2 × 10^11^ cm^−2^, 0, and −7.2 × 10^11^ cm^−2^. The position of the local Dirac point peak of graphene FET does not change with decreasing temperature indicating the immobilization of commensurate ferroelectric domains of *t*‐WSe_2_ with temperature. Moreover, a crossover in the temperature‐dependent *R_xx_* vs *n_B_* curves at particular *n_B_* or at critical carrier density $n_{B}^{C}$ indicates multiple MITs in the *R*_xx_ vs *T* characteristic of graphene FETs when coupled with ferroelectric *t*‐WSe_2_ (Figure S3, Supporting Information).

**Figure 3:**
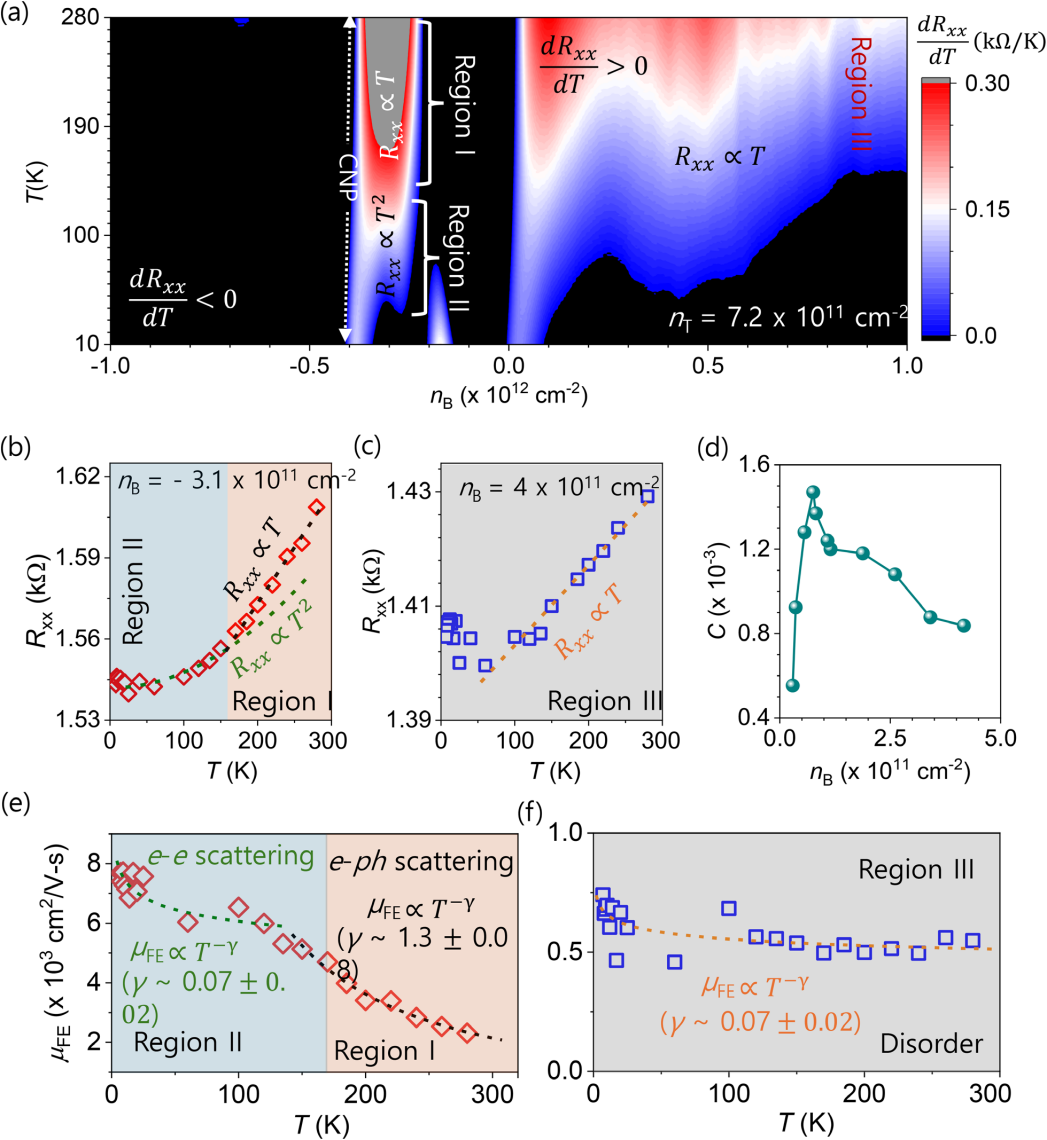
Doping‐driven distinct metallic phases: a) 2D color map of the first derivative of resistance, $\frac{dR_{xx}}{dT}$ as a function of *n_B_* and temperature for fixed *n_T_* = +7.2 × 10^11^ cm^−2^, indicating distinct metallic ($\frac{dR_{xx}}{dT}>0$) and insulating ($\frac{dR_{xx}}{dT}<0$) phases. b) In the metallic phase regime, (−2.2 × 10^11^ < *n_B_* < −3.95 × 10^11^ cm^−2^), where $\frac{dR_{xx}}{dT}>0$, *R_xx_* exhibits a *T* ^2^ dependence at low temperature (10–100 K, region II), which is a characteristic of Landau Fermi‐liquid phase suggesting the dominant scattering mechanism arises from weak electron‐electron interactions. At high temperatures (*T* > 150 K, Region I), the *T*‐linear dependence of *R*_XX_ is attributed to phonon scattering. c) For *n_B_* > 0 (Region III), *R_xx_* follows a linear *T*‐dependence over a wide range of temperatures from 50 to 280 K. In this regime, carrier transport is primarily governed by disorder scattering. d) The variation of the numerical pre‐factor *C* of the transport scattering rate $\Gamma (\Gamma =C\frac{k_{B}T}{\hbar })$ with *n_B_*. Here, *C* varies from ≈0.0012 to ≈0.0008 which is significantly lower than the value for Planckian dissipation, suggesting the dominant role of disorder. e, f) Variation of field‐effect mobility, *µ*_FE_ with temperature when measured near the CNP and *n*_B_≫0 respectively. The dashed lines represent the corresponding fits of the µ_*FE*_ to µ_*FE*_ ∝ *T* ^−γ^, where *γ* is the mobility exponent. A lower value of *γ* indicates a weaker (nearly temperature‐independent) mobility, whereas a higher value of *γ* corresponds to a stronger temperature dependence of µ_*FE*_.

Doping‐driven distinct metallic phases, $\frac{dR_{xx}}{dT}>0$: **Figure** **3**a shows the 2D color map of the first derivative of *R*_xx_, i.e., $\frac{dR_{xx}}{dT}$ as a function of *n_B_* and *T* for fixed *n_T_* = 7.2 × 10^11^ cm^−2^, which manifests distinct metallic ($\frac{dR_{xx}}{dT}>0$) and insulating ($\frac{dR_{xx}}{dT}<0$) phases of the *t*‐WSe_2_/graphene vdW heterostructure for *θ* = 1.5°. The vertical white arrow indicates the CNP at *n_B_* = −4.8 × 10^11^ cm^−2^ because the fixed *n*_T_ of 7.2 × 10^11^ cm^−2^ results in holes being the majority carriers in monolayer graphene. Figure S4 (Supporting Information) in the Supporting Information reveals similar insulating and metallic phases for *n_T_* of −7.2 × 10^11^ cm^−2^, and 0, respectively. It is visualized that the MIT is highly impacted by *n_B_*. For higher negative *n_B_* (electron regime), we observed the insulating phase for the whole temperature range from 10 to 280 K. However, in the positive *n_B_* region (hole regime), we observed MIT in the temperature ranges from 110 to 40 K depending upon electrostatic doping, *n_B_*. Additionally, another insulating phase is observed for *n_B_* between 0 and −2.1 × 10^11^ cm^−2^.

These intriguing metallic and insulating regimes as a function of *n_B_* can be explained using the dimensionless interaction parameter, *r*_s,_ which represents the ratio of Coulomb energy (*E*_C_) to kinetic energy (*E*_F_) at the Fermi level.^[^ ^10^ ^]^ It is defined as, *r*_s_ = *E*_C_/*E*_F_ =$m^{*}\;e^{2}n_{V}/4\pi \varepsilon \hbar^{2}\sqrt{\pi n}$, where *n_V_* is the number of valleys and *n* is the total carrier density at the MIT points. At low doping concentration *n*, the suppression of *E*_F_ with respect to *E*_C_ results in insulating behavior. Conversely, at higher doping level, *E*_F_ surpasses *E*_C_, leading to metallic behavior. However, the metallic region (I and II), the insulating regime between 0 and −2.1 × 10^11^ cm^−2^, and the MIT at higher doping levels cannot be fully explained by the dimensionless interaction parameter, *r*_s_. Instead, they appear to exhibit a periodic variation of metal and insulator phases with doping, which may be attributed to the variation of *m* ^*^ with *D*. Mann *et* al. reported that *m* ^*^ is modulated with *D* in trilayer graphene, modifying $r_{s}\propto m^{*}/\sqrt{n}$ accordingly.^[^ ^31^ ^]^ At higher doping level, the *n_B_* dependence of the MIT temperature is also influenced by the modulation of m* with *D*, affecting transport properties and requires further investigation. Additionally, the MIT is also influenced by *n_T_*, indicating a dynamical interplay between commensurate ferroelectric domains and *n_T_*, which modulates the electronic structure of monolayer graphene. (Figure S4, Supporting Information).

**Figure 4:**
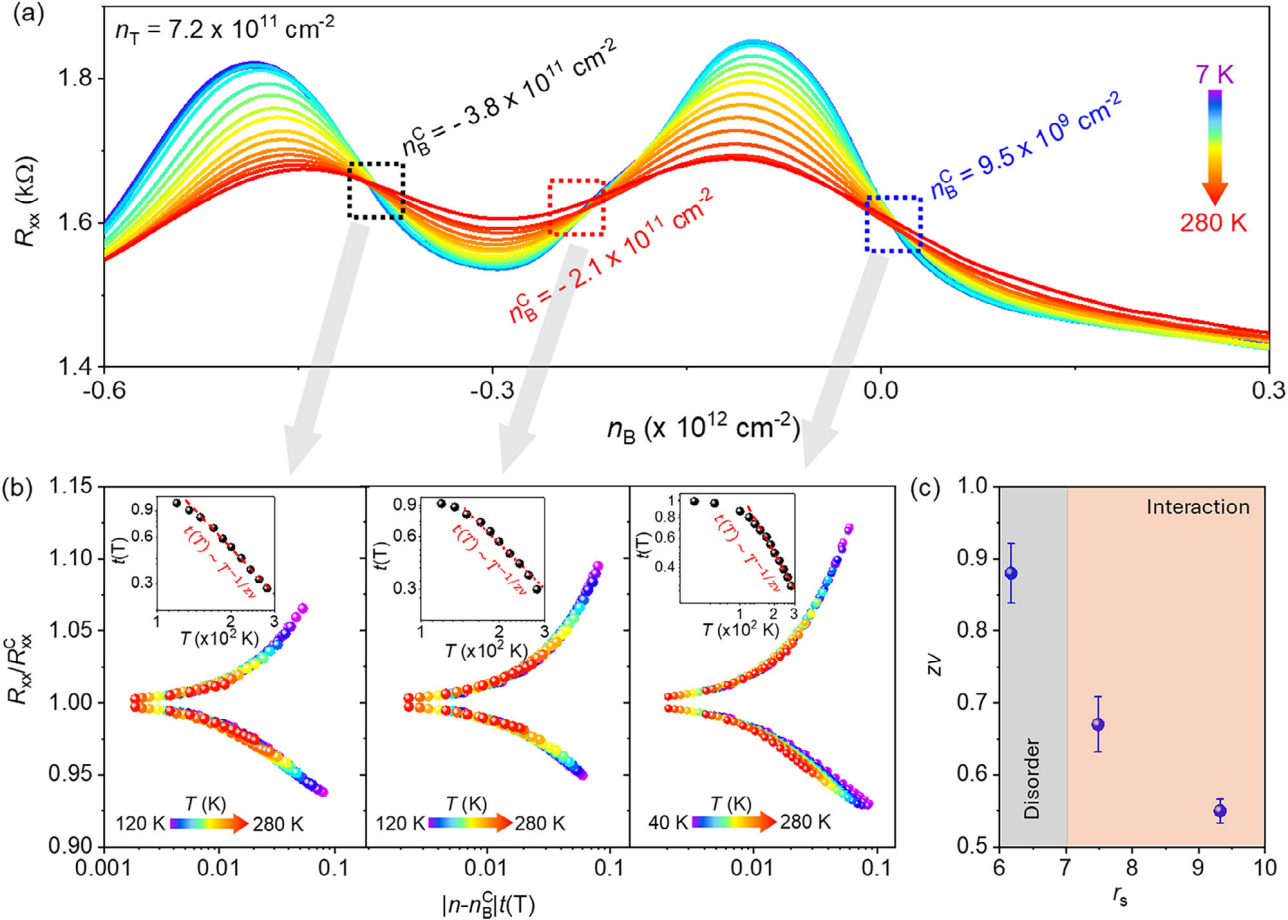
Quantum critical behavior of MITs: a) Temperature‐dependent *R*_xx_ vs *n_B_* curves over the temperature range from 7 to 280 K revealing multiple MIT points at $n_{B}^{c}$ = −3.8 × 10^11^ cm^−2^, −2.1 × 10^11^ cm^−2^ and 9.5 × 10^9^ cm^−2^ while *n_T_* is fixed at 7.2 × 10^11^ cm^−2^. b) The plots of $R_{xx}\left(\;T\right)/R_{xx}^{c}\left(T\right)$ vs $|n-n_{B}^{C}|t\left(T\right)$ at various temperatures near MIT points. Insets show that *t*(*T*) follows a power‐law dependence on temperature as *t*(*T*)  ≈  *T* ^−1/*zv*^, indicating multiple continuous quantum phase transitions in graphene/*t*‐WSe_2_ vdW heterostructures. c) The obtained values of critical exponents at different MIT points decrease with increasing dimensionless interaction parameter, *r*_s_.

We now analyze the behavior of *R*_xx_ vs *T* curves at various *n_B_* values, both near and away from the crossover points, $n_{B}^{C}$. The nearly temperature independent *R*_xx_ at *n_B_* = 9.5 × 10^9^ cm^−2^, −2.1 × 10^11^ cm^−2^ and −3.8 × 10^11^ cm^−2^ is a signature of $n_{B}^{C}$ for the MIT (Figure S5, Supporting Information). Four features are observed in the *R*_xx_ vs *T* characteristics for both the electron‐ and hole‐doped regimes. First, in the insulating phase regime (−2.1 × 10^11^ cm^−2^ < *n_B_* < 0) where $\frac{dR_{xx}}{dT}<0$, *R*_xx_ follows $R_{xx}\propto e^{{\left(\frac{T_{ES}}{T}\right)}^{x}}$ with $x=\frac{1}{2}\;$, where *T_ES_* is the hopping parameter (Figure S6, Supporting Information). This is consistent with the Efros‐Shklovskii variable range hopping model, providing evidence that *E*_C_ plays a significant role in the insulating phase for *n_B_* between 0 and −2.2 × 10^11^ cm^−2^.^[^ ^10^, ^32^ ^]^ Second, in the metallic phase regime (−2.2 × 10^11^ < *n_B_* < −3.8 × 10^11^ cm^−2^), where $\frac{dR_{xx}}{dT}>0$, *R_xx_* exhibits linear *T*‐dependence of *R*_xx_ at high temperature (*T* > 150 K, Region I) and *T* ^2^ dependence at low temperature (10–100 K, Region II) indicating distinct scattering mechanisms for carrier transport at different temperature ranges (Figure 3b). The *T^2^* and *T*‐linear dependence of *R*_xx_ with temperature at particular electrostatic doping, indicative of Fermi liquid and non‐Fermi liquid metallic behavior, respectively. Third, for *n_B_* < −3.5 × 10^11^ cm^−2^, we have observed the characteristic of a band insulator near the CNP of graphene. However, even far from the CNP for *n_B_* ≪ −4.8 × 10^11^ cm^−2^, insulating behavior persists. This is attributed to a decrease in the majority carrier concentration at higher doping levels, causing *R_xx_* to follow a thermally activated dependence. (Figure S6, Supporting Information). Fourth, in the metallic regime for *n_B_* > 0, an *n_B_* dependent MIT is observed, and *R_xx_* follows a linear *T*‐dependence over a wide temperature range from 50 to 280 K (**Figure** **4**c; Figure S6, Supporting Information).

The *T*‐linear dependence of *R*_xx_ could arise from strong electron‐electron interactions at the *t*‐WSe_2_/graphene interface and is often associated with the concept of Planckian dissipation. In Planckian dissipation, the scattering rate, $\Gamma (\Gamma =\frac{Ck_{B}T}{\hbar }\;)$ is limited solely by temperature in the vicinity of the quantum critical point, where the numerical pre‐factor *C* (=$\frac{\hbar e^{2}n}{m^{*}k_{B}}\;a$) approaches ≈1. Here, ℏ is the reduced Planck constant, *e* is the electronic charge, *k*_B_ is the Boltzmann constant, *α* is the slope of the *R*_xx_ vs *T* curves when linearly fitted with *R_xx_* (*T*) =  α*T* + β, and β is the residual resistance. For *t*‐WSe_2_, the effective mass is ≈ 0.4 *m_e_* and *n*  =  1.47 × 10^12^ cm^−2^, then $C=\frac{\hbar e^{2}n}{k_{Bm^{*}}}\;\alpha \approx 0.027\alpha$.^[^ ^9^, ^10^, ^33^ ^]^ In our case, *C* varies from ≈0.0012 to ≈0.0008, which is significantly lower than the expected value for Planckian dissipation. This indicates the absence of strong electron‐electron correlation within the measured temperature and *n_B_* range (Figure 3d). Therefore, the scattering mechanism in our device is likely influenced by weak electron‐electron scattering along with other factors such as phonons, disorders, impurities, etc.

In this context, we plot the field‐effect mobility, *µ*_FE_, as evaluated from different regions with respect to *n*_B_ (Figure 3e,f). The temperature‐dependent behavior of *µ*_FE_ varies across different regions. In the metallic regime (−2.2 × 10^11^ < *n_B_* < −3.8 × 10^11^ cm^−2^), *µ*_FE_ decreases with increasing temperature, suggesting the electron‐phonon scattering(*T* > 100 K, Region I). In contrast, *µ*_FE_ saturates at lower temperatures (*T* < 100 K, Region II), indicating that either the electron‐electron scattering or electron‐impurity scattering dominates over the electron‐phonon scattering. Further, we fit mobility to µ_*FE*_ ∝ *T* ^−γ^,^[^ ^34^ ^]^ where γ is the mobility exponent. The extracted lower value of γ = 0.07 ± 0.02 confirms the temperature‐independent *µ*_FE_ in Region II. The high and temperature‐independent mobility supports the electron‐electron scattering that leads to the *T* ^2^‐ dependence of *R*_xx_ at low temperatures (10–150 K). In region I, the extracted value of γ = 1.3 ± 0.02 confirms that *µ*_FE_ strongly depends on temperature, leading to a linear *T*‐dependence of *R*_xx_ at high temperatures owing to the electron‐phonon scattering (*T* > 150 K).

For *n*_B_ ≫ 0, *µ*_FE_ remains constant (γ = 0.07 ± 0.02) across the entire temperature range (Region III). This may arise from electron‐electron scattering or from the presence of disorders in the conducting channels. However, the observed *µ*_FE_ is one order of magnitude lower compared to the *µ*_FE_ observed for electron‐electron scattering (Region II), indicating that disorder plays a dominant role here. The presence of this disorder leads to the observed linear‐*T* dependence of *R*_xx_ in the hole doped regime.

Quantum critical behaviors of the metal‐insulator transitions: To examine the quantum critical behaviors near the MITs, we performed a finite‐size scaling (FSS) analysis of *R*_xx_ in the vicinity of the MIT points at $n_{B}^{c}$ = −3.8 × 10^11^ cm^−2^, −2.1 × 10^11^ cm^−2^ and 9.5 × 10^9^ cm^−2^ while *n_T_* is fixed at 7.2 × 10^11^ cm^−2^ (Figure 4a). According to FSS, we express *R*_xx_ in terms of *T* and *n_B_* as $R_{xx}\;\left(n_{B},T\right)=R_{xx}^{c}\;F\left[|n-n_{B}^{C}|t\left(T\right)\right]$,^[^ ^35^ ^]^ where *F* represents a universal scaling function, $R_{xx}^{c}$ signifies the resistance at the $n_{B}^{c}$ (or at the MIT points), and *t*(*T*)  ≈  *T* ^−1/*zv*^, where *z* and ν stand for the dynamical exponent and correlation length, respectively. Figure 4b shows the plots of $R_{xx}\left(\;T\right)/R_{xx}^{c}\left(T\right)$ vs $|n-n_{B}^{C}|t\left(T\right)$ at various temperatures near MIT points. Here, *t*(*T*) is optimized to collapse the different constant‐temperature data onto a single curve. Further, analysis shows that *t*(*T*) follows a power‐law dependence on temperature as *t*(*T*)  ≈  *T* ^−1/*zv*^, yielding the critical exponents at different MIT points (insets of Figure 4b). The obtained values of critical exponents are *z*ν  =  0.55 ± 0.02, *z*ν  =  0.67 ± 0.04, and *z*ν  =  0.88 ± 0.04 for $n_{B}^{c}$ = −3.8 × 10^11^ cm^−2^, ‐2.1 × 10^11^ cm^−2^ and 9.5 × 10^9^ cm^−2^, respectevely, demonstrating multiple continuous quantum phase transistions (QPTs) between metallic and insulating phases within the temperature range of 120–280 K in monolayer graphene coupled with *t*‐WSe_2_ moiré ferroelectric.

It is interesting to note that the critical exponent *z*ν strongly depends on dimensionless interaction parameter, *r*_s_. Figure 4c shows that the obtained values of critical exponents increase with decreasing *r*_s_. Here, we fixed the *m* ^*^ value as ≈0.4. The lower values of critical exponent *z*ν  ≈ 0.5–0.7 along with high‐*r*_s_ is consistent with that observed for a continuous QPTs from a Wigner crystal to a strange metal in a strongly correlated chiral‐stacked twisted double bilayer graphene vdW heterostructure (*z*ν  ≈  0.5),^[^ ^32^ ^]^ dynamical mean‐field theory extracted critical exponents *z*ν  =  0.56 −0.57,^[^ ^36^ ^]^ experimentally reported critical exponents *z*ν  =  0.49 −0.68 for Mott organic materials,^[^ ^37^ ^]^ *z*ν  ≈  0.7 for MoTe_2_/WSe_2_ moiré superlattice at fillings where the strongly correlated regime dominated by Mott‐like physics,^[^ ^8^ ^]^ and supporting the interaction‐driven QPT in *t*‐WSe_2_/graphene at high‐*r*_s_ values. On the other hand, the larger value of critical exponent *z*ν  =  0.88 ± 0.04 along with low‐*r*_s_ closely matches the critical exponent *z*ν  =  1 ± 0.1 of MoTe_2_/WSe_2_ moiré superlattice at fillings far from the strongly correlated regime,^[^ ^38^ ^]^ suggesting the disorder‐driven continuous QPTs in *t*‐WSe_2_/graphene.

## 3 Conclusion

We demonstrated a MIT near room temperature in monolayer graphene, arising from sublattice symmetry breaking induced by the periodic potential imprinted by *t*‐WSe_2_. The emergence of a local Dirac point in the transport characteristics of graphene coupled with moiré ferroelectricity, provides direct evidence of symmetry breaking. Temperature‐dependent transport measurements revealed multiple MITs driven by electrostatic doping. These transitions were associated with the dynamical interplay between the ferroelectric order of *t*‐WSe_2_ and the electronic structure of graphene, leading to distinct scattering regimes characterized by *T^2^* – and linear *T*‐dependent resistivity, indicative of Fermi‐liquid and non‐Fermi‐liquid metallic behavior, respectively. Finite‐size scaling analysis of the *R*_xx_ near the MIT points indicate continuous QPTs, establishing a platform for exploring tunable QPTs and correlated electron phenomena in vdW heterostructures.

## 4 Experimental Section

### Device Fabrication and Transport Measurements

The twisted vdW heterostructure was fabricated using the widely recognized tear‐and‐stack technique. Monolayer graphene, WSe_2_, and *h*‐BN flakes were mechanically exfoliated onto O_2_ plasma‐treated (5 sccm) SiO_2_/Si substrates. The substrates were subjected to heat treatment at 100 °C for 10 min and inspected using optical microscopy. The WSe_2_ single crystals and kish graphite used for exfoliating monolayer graphene were sourced from high‐quality commercial suppliers (HQ Graphene and 2D Semiconductors).

A stack of poly (bisphenol A carbonate) (PC) and polydimethylsiloxane (PDMS) was mounted on a glass slide and positioned on a micro‐positioning auto‐transfer stage inside a glove box with controlled atmosphere (O_2_ and H_2_ < 0.5 ppm). In this environment, a *h*‐BN flake was picked up at 110 °C. The *h*‐BN was then used to tear a monolayer WSe_2_ flake at 100 °C. The torn WSe_2_ halves were rotated manually by an angle of *θ* = 1.5° (to 5°) and re‐stacked. Monolayer graphene was subsequently picked up using the *h*‐BN/*t*‐WSe_2_ structure and encapsulated by adding a bottom *h*‐BN layer. The top *h*‐BN layer (10–30 nm) served as the top gate dielectric, while the SiO_2_ layer (290 nm) acted as the bottom gate dielectric. The complete stack was released onto a SiO_2_/Si substrate at 220 °C. The final device geometry was defined using electron‐beam lithography and inductively coupled plasma etching.

All transport measurements were performed in a low‐temperature cryostat (Cryomagnetics Inc., Oak Ridge, USA) equipped with a maximum 9 T magnetic field. Electrical signals were measured using an SR830 lock‐in amplifier, and gate voltages (top and bottom) were applied using a Keithley 2400 source meter.

## Conflict of Interest

The authors declare no conflict of interest.

## Author Contributions

BS and YH contributed equally to this work. B.S., P.K.S. and C.L. conceived the project. Y.H. and T.D.N. prepared the devices for electrical transport measurements. B.S., P.K.S. and Y.H. conducted the electrical measurements. W.J.Y. assisted with the glove box facility and its operation. K.W. and T.T. provided the *h*‐BN single crystals. B.S., P.K.S., N.A. and C.L. analysed the data and co‐wrote the manuscript. S.D., J.J., J.S., M.S.A., M.S.C., T.K., H.M.J., S.L. and S.G. contributed to the experimental measurements and manuscript writing.

## Supporting information



Supporting Information

## Data Availability

The data that support the findings of this study are available from the corresponding author upon reasonable request.
