# Supplementary material for: Quantum Phase Transitions in Graphene Coupled to a Twisted WSe2 Moiré Ferroelectricity
Source: Adv Mater. 2025 Oct 22;38(5):e14744. doi: 10.1002/adma.202514744 (PMC12822538; doi:10.1002/adma.202514744)
Supplement: Supplementary file 1 — Supporting Information [file ADMA-38-e14744-s001.docx]

**Supporting Information**

**Quantum Phase Transitions in Graphene Coupled to a Twisted WSe_2_ Moiré Ferroelectricity**

Budhi Singh^1‡^,Yasir Hassan^2‡^, Nasir Ali^1^, Santosh Durairaj^3^, Jimin Jang^1^, Tien Dat Ngo^1^, Jyoti Saini,^4^ Muhammad Sabbtain Abbas^5,6^, Kenji Watanabe^7^, Takashi Taniguchi^7^_,_ Min Sup Choi^2^, Subhasis Ghosh^4^, Taesung Kim^3^, Hyung Mo Jeong^3^, Sungjoo Lee^1^, Won Jong Yoo^1^, Pawan Kumar Srivastava^3,8,9*^, Changgu Lee^1,3*^

^1^SKKU Advanced Institute of Nanotechnology (SAINT), Sungkyunkwan University, Suwon

16419, South Korea

^2^Department of Materials Science and Engineering, Chungnam National University, 99, Daejeon, South Korea

^3^School of Mechanical Engineering, Sungkyunkwan University, Suwon, 16419, South Korea

^4^School of Physical Sciences, Jawaharlal Nehru University, New Delhi 110067, India

^5^Insitute of Applied Physics, Seoul National University, Seoul, South Korea

^6^Centre for Advanced Studies in Physics, GC University Lahore, Pakistan 54000

^7^National Institute for Materials Science, Namiki 1-1, Tsukuba, Ibaraki 305-0044, Japan

^8^Center for 2D Quantum Heterostructures, Institute for Basic Science (IBS), Sungkyunkwan

University (SKKU), Suwon, Republic of Korea-16419

^9^Department of Physics, National University of Singapore, Science Drive 3, Singapore 117542

‡These authors contributed equally

^*^Corresponding authors: [pksri08@gmail.com](mailto:pksri08@gmail.com); peterlee@skku.edu

**S1. Transfer characteristics of t-WSe_2_/graphene vdW heterostructure**


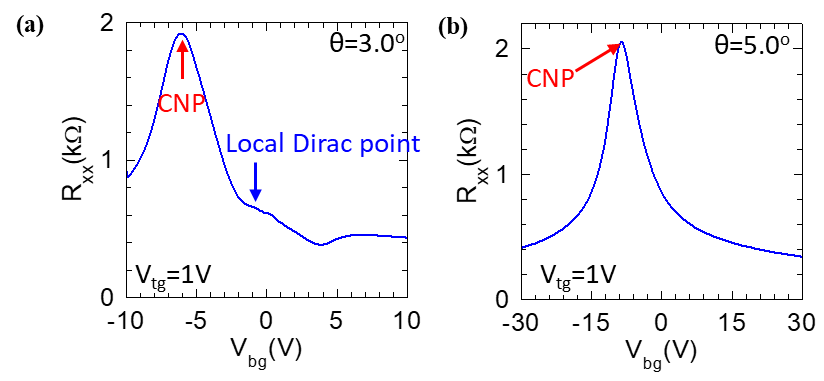


**
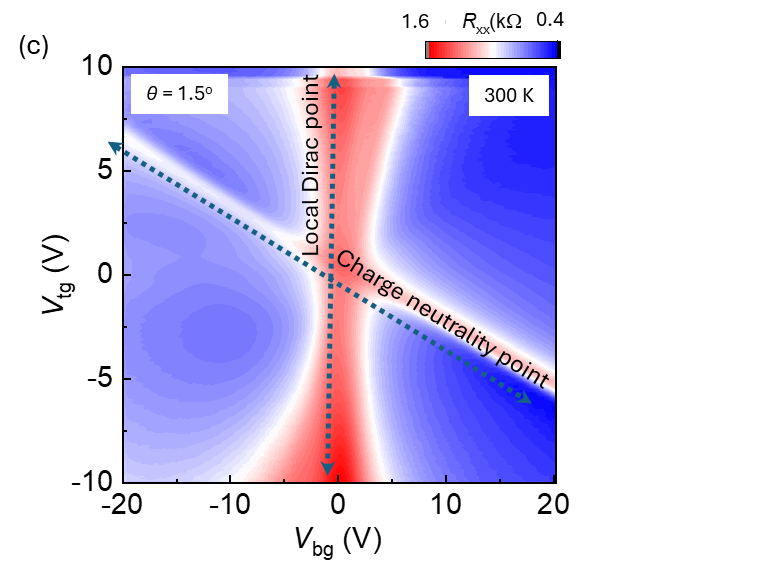
**

**Figure S1:** Transfer characteristics of *t*-WSe₂/graphene vdW heterostructures for *θ* = **(a)** 3^o^ and **(b)** 5^o^ at room temperature with top gate voltage *V*_tg_=1 V. Due to the presence of commensurate ferroelectric domain-induced local Dirac points, an additional peak appears in the transfer characteristics of the graphene FET for *θ*=3°, although it is not as prominent as for *θ*=1.5° (as shown in the Figure S1c and main text). At a higher twist angle, *θ*=5.0°, no such peak is observed, as there are no commensurate ferroelectric domains, leading to the absence of hysteresis in the transfer characteristics of the graphene FET.

**S2. Energy level band alignment in t-WSe_2_/graphene vdW heterostructure**


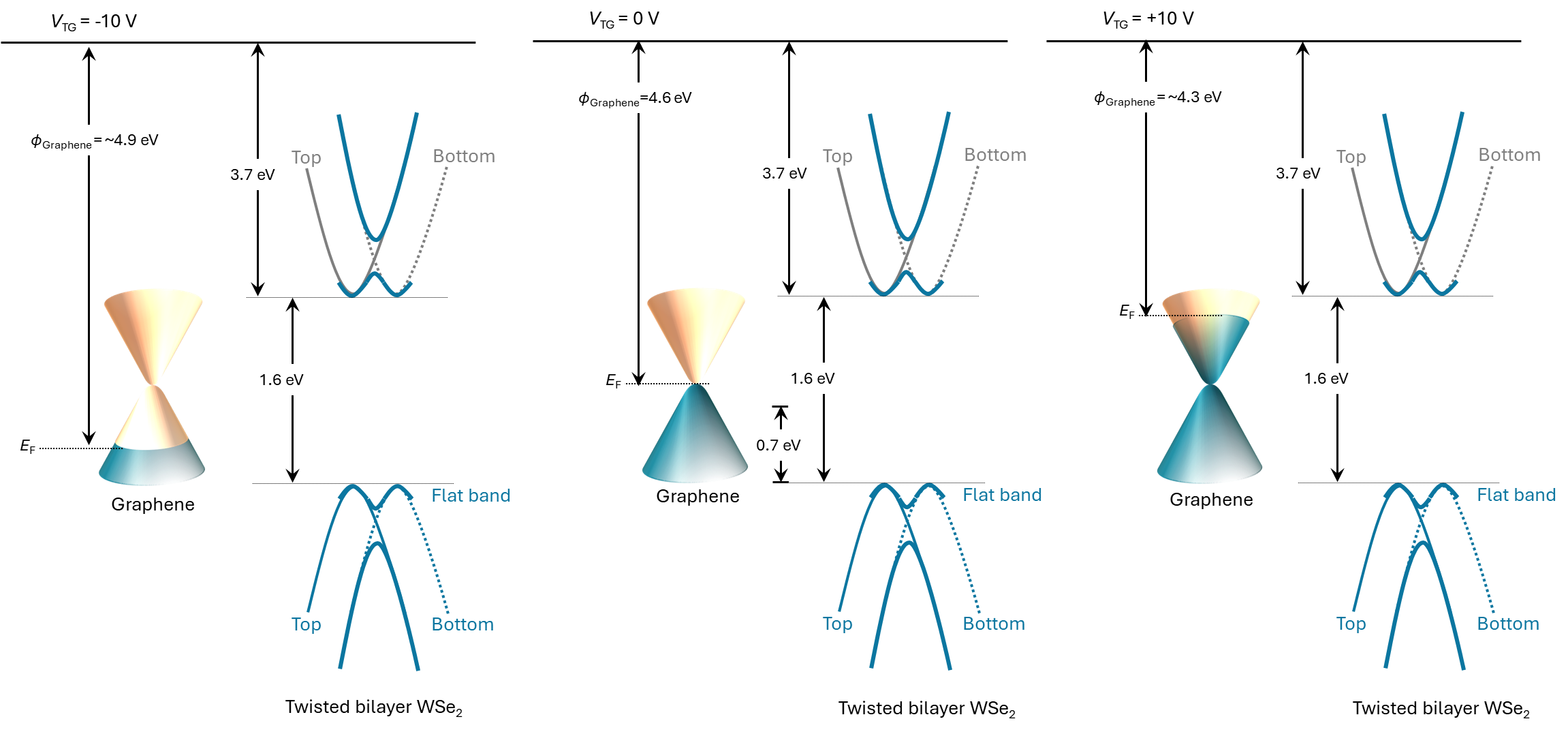


**Figure S2:** Band alignment in the *t*-WSe_2/_graphene vdW heterostructure for 0 and ±*V*_TG_​, (=±10 V). The top gate voltages only shift the Dirac point maximum up 0.1-0.3 eV as per our device capacity before the *h*-BN dielectric breakdown. The maximum Fermi energy of the graphene is~ 4.9 eV, therefore, the back gate doped carrier will enter in the graphene and cannot be transferred into the WSe_2_. This is due to the large energy difference (> 0.5 eV) between the graphene Dirac point (~4.6 eV) and the WSe_2_ band edges (~5.31 eV).

**S3. Metal insulator transition**

**
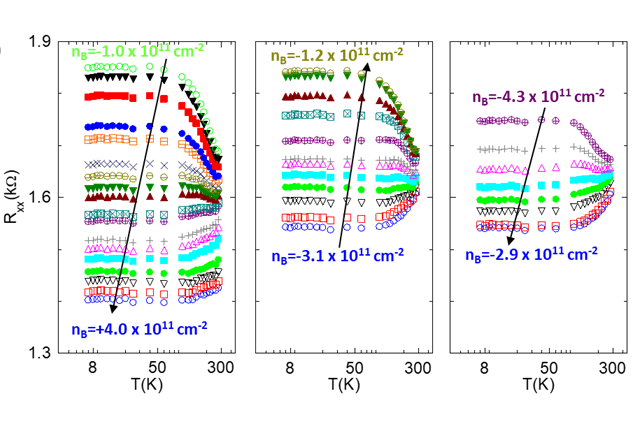
**

**Figure S3:** (a) $R_{xx}$ *vs* *T* curves within the temperature range from 8 K to 280 K at various $n_{B}$ near the MIT crossover points at $n_{B}$ =~ +1.0 x 10^10^ cm^-2^, -2.2 x 10^11^ cm^-2^ and -3.95 x 10^11^ cm^-2^ when $n_{T}$ is fixed at +7.2 x 10^11^ cm^-2^.

**S4. 2D color map of the** $\frac{\boldsymbol{d}\boldsymbol{R}_{\boldsymbol{xx}}}{\boldsymbol{dT}}$ **as a function of** $\boldsymbol{n}_{\boldsymbol{B}}$ **and temperature**


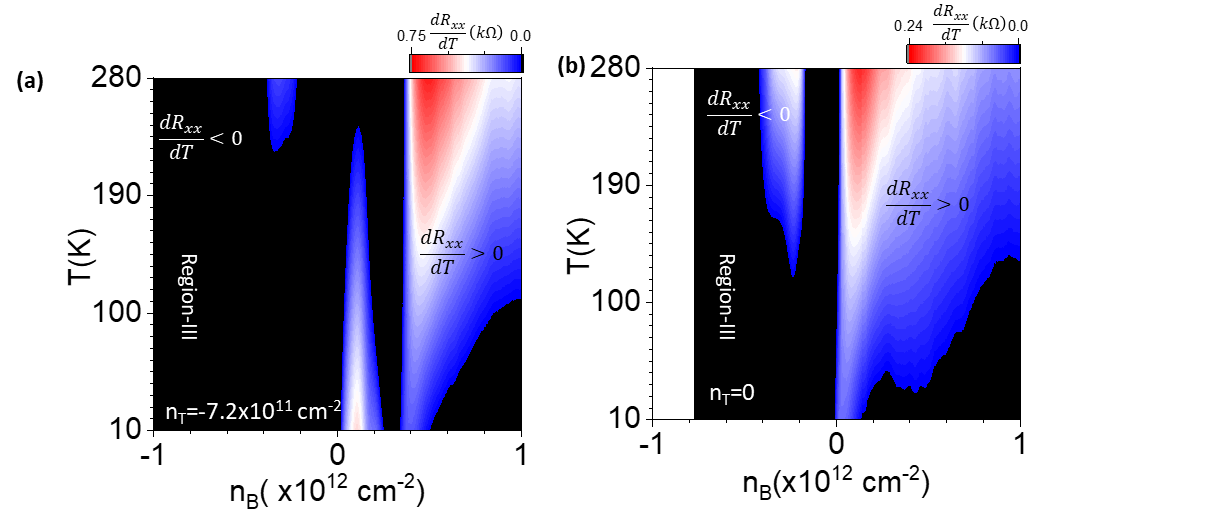


**Figure S4**: Two- dimensional color map of the first derivative of resistance, $\frac{dR_{xx}}{dT}$ as a function of $n_{B}$ and temperature for fixed **(a)**$n_{T}$ = -7.2 x 10^11^ cm^-2^, and **(b)** $n_{T}$ = 0 indicating distinct metallic ($\frac{dR_{xx}}{dT}>0$) and insulating ($\frac{dR_{xx}}{dT}<0$) phases (*θ*=1.5^o^).

**S5. Temperature dependent** $\boldsymbol{R}_{\boldsymbol{xx}}$ **at critical carrier concentration,** $\boldsymbol{n}_{\boldsymbol{B}}^{\boldsymbol{C}}$


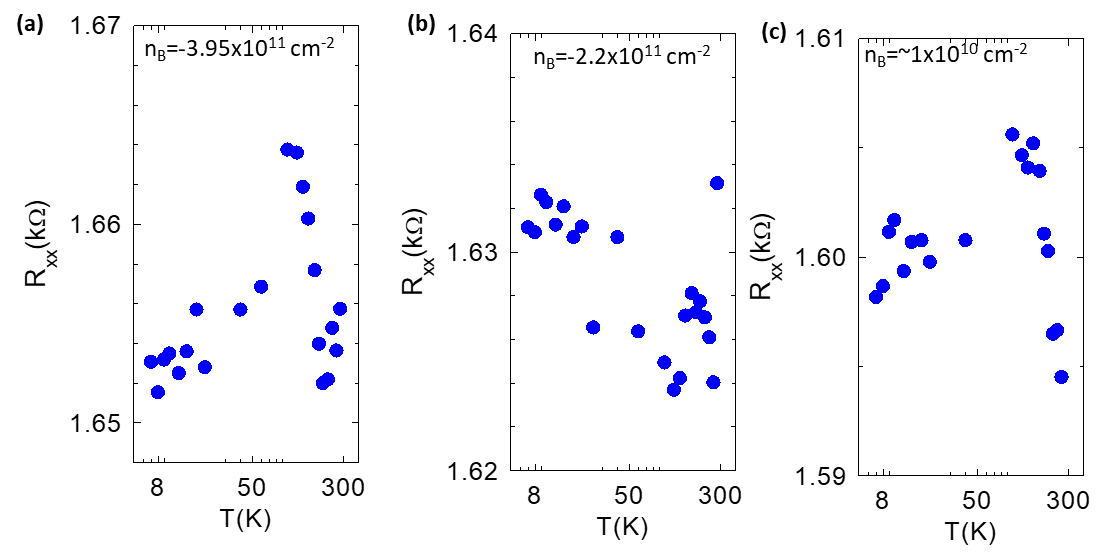


**Figure S5:** Almost temperature independent $R_{xx}$at $n_{B}$**(a)** -3.95 x 10^11^ cm^-2^ **(b)** $n_{B}$ = -2.2 x 10^11^ cm^-2^, and **(c)** 1 x 10^10^ cm^-2^ is a signature of critical carrier concentration,$n_{B}^{C}$ for MIT.

**S6. Temperature dependent** $\boldsymbol{R}_{\boldsymbol{xx}}$ **at different** $\boldsymbol{n}_{\boldsymbol{B}}$ **for** $\boldsymbol{n}_{\boldsymbol{T}}$ **= 7.2 x 10^11^ cm^-2^**

**
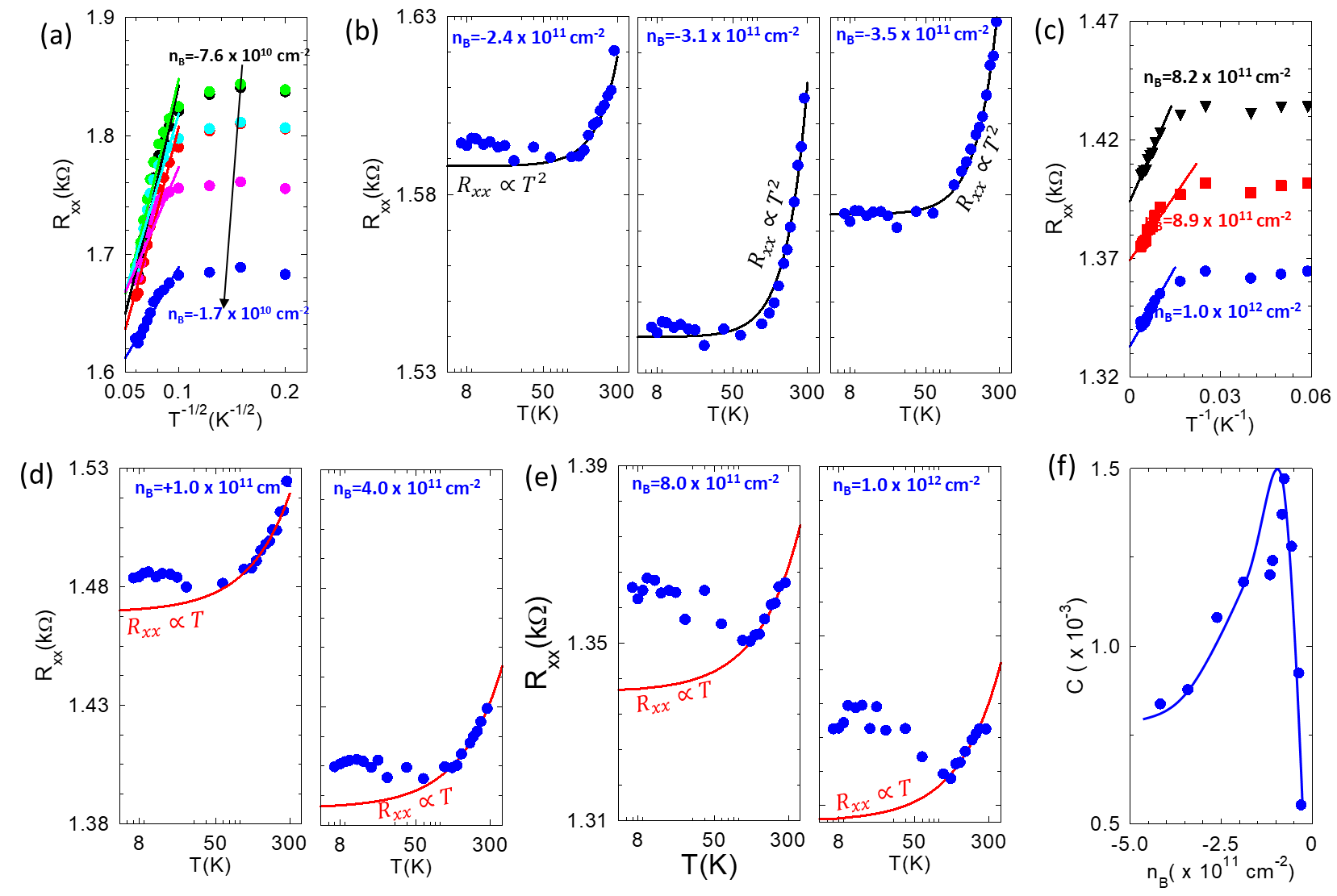
**

**Figure S6: : Electron doped regime (a)** In the insulting phase ($\frac{dR_{xx}}{dT}<0$, Region II), $R_{xx}$ follows the Efros-Shklovskii variable range hopping model, providing evidence that the Coulomb interaction in the insulating phase of the electron regime. **(b)** In the metallic phase, where $\frac{dR_{xx}}{dT}>0$, $R_{xx}$ behavior revealed $T^{2}$ dependence which is a characteristic of Landau Fermi liquid phase. **(c)** In the insulting phase ($\frac{dR_{xx}}{dT}<0$, Region III), $R_{xx}$follows thermal activation dependence, indicating the suppressed hopping of localized electrons at higher electron doping. **Hole doped regime (d-e)** In the metallic phase, where $\frac{dR_{xx}}{dT}>0$, $R_{xx}$ follows a linear $T$ dependence over a wide range of temperatures from 50 K to 280 K . **(f)** The variation of the numerical pre-factor $C$ of the transport scattering rate $\Gamma\left( \Gamma=\frac{Ck_{B}T}{\hbar} \right)$ with $n_{B}$. Here, $C$ varies from ~0.0012 to ~0.0008 which is significantly lower than the value for Planckian dissipation indicates the contribution of electron phonon scattering.

**S7. Ruling out the contribution from the disorder, interfacial strain, or partial hybridization at the *t*-WSe_2_/graphene interface**

1. **Ruling out disorder**
2. If disorder were dominant, additional *R*_xx_ peaks would appear randomly and vary by sample. Instead, we observe a systematic dependence on the ferroelectric polarization in *t*-WSe_2_ (*Figure 1 of the main manuscript*).
3. Ferroelectric polarization in *t*-WSe_2_ can only imprint onto graphene with a clean, disorder-free interface. Multiple forward/backward gate scans confirm that the ferroelectricity is robust and not due to traps or impurities, indicating a defect-free interface (Ref. [15] of the manuscript)**.**
4. **Ruling out lattice mismatch**
5. Unlike conventional thin films, WSe_2_ and graphene are separated by a vdW gap, minimizing lattice mismatch effects.
6. Graphene/WSe_2_ interfaces exist in all devices regardless of ferroelectric polarization. Since careful alignment is only applied between WSe_2_ bilayers (not graphene/WSe_2_), lattice mismatch would affect all devices equally and not depend on the WSe_2_ twist angle.
7. **Ruling out hybridization**
8. Hybridization strength is set by the graphene/WSe_2_ interface; the twist angle between WSe_2_ bilayers does not directly affect the graphene/WSe_2_ separation, hence, orbital overlap with graphene.
9. If hybridization dominated, transport should be similar across all samples (as graphene/WSe_2_ separation at the interface remains unchanged). Instead, gate-tunable transport strongly depends on the WSe_2_ bilayer twist angle.
10. Position of Dirac point remains at *V*_G_ ~ 0V, in both, *h*-BN (Ref. [15] of the manuscript) and *t*-WSe_2_ encapsulated graphene (*see* *Figure 1 of the manuscript*), ruling out any possible hybridization between graphene and WSe_2_.
